# Preverbal infants expect agents exhibiting counterintuitive capacities to gain access to contested resources

**DOI:** 10.1038/s41598-021-89821-0

**Published:** 2021-05-25

**Authors:** Xianwei Meng, Yo Nakawake, Kazuhide Hashiya, Emily Burdett, Jonathan Jong, Harvey Whitehouse

**Affiliations:** 1 Faculty of Human-Environment Studies, Kyushu University, Fukuoka, Japan; 2 Graduate School of Human Sciences, Osaka University, Suita, Japan; 3 Center for Baby Science, Doshisha University, Kyoto, Japan; 4 School of Economics and Management, Kochi University of Technology, Kochi, Japan; 5 Centre for the Study of Social Cohesion, University of Oxford, Oxford, UK; 6 Belief, Brain and Behaviour, Coventry University, Coventry, UK; 7 School of Psychology, University of Nottingham, Nottingham, UK

**Keywords:** Evolution, Anthropology, Cultural evolution, Social evolution, Psychology, Human behaviour, Zoology, Animal behaviour

## Abstract

Claims to supernatural power have been used as a basis for authority in a wide range of societies, but little is known about developmental origins of the link between supernatural power and worldly authority. Here, we show that 12- to 16-month-old infants expect agents exhibiting counterintuitive capacities to win out in a two-way standoff over a contested resource. Infants watched two agents gain a reward using either physically intuitive or physically counterintuitive methods, the latter involving simple forms of levitation or teleportation. Infants looked longer, indicating surprise, when the physically intuitive agent subsequently outcompeted a physically counterintuitive agent in securing a reward. Control experiments indicated that infants’ expectations were not simply motived by the efficiency of agents in pursuing their goals, but specifically the deployment of counterintuitive capacities. This suggests that the link between supernatural power and worldly authority has early origins in development.

## Introduction

Positions of authority in human societies may derive, at least in part, from claims to supernatural power^1,2^. Examples range from diviners, shamans, and witch-doctors in small-scale societies^3–5^ to god-kings of archaic states^6^ and the divinely sanctioned powers of rulers of Axial Age social formations and the world religions they spawned^7,8^. Little is known, however, about the nature of the link between authority and supernatural power, and still less about its developmental origins.

Supernatural power attribution commonly entails capacities to efficiently achieve goals by counterintuitive methods violating intuitive physics (e.g., levitation), biology (e.g., healing) and psychology (e.g., mind reading)^9–12^. Supernatural powers based on physically counterintuitive capacities feature frequently in folktales and myths. For example, the motif of “magical transportation” can be found in Eskimo, Icelandic, Indian, Irish, Jewish and Spanish myths and folktales, while the motif of “human levitation” has been recorded in Irish, Jewish, Hindu and Indo-Chinese Buddhist traditions^13–15^. In many of these cultures, individuals attributed with such capacities occupy higher social status allowing them to exercise authority in worldly matters. The current study investigates the developmental origin of the link between social dominance and physically counterintuitive capacities by testing whether infants expect agents who effectively achieve goals by means violating intuitive physics to gain access to contested resources. Although there is evidence of an early emerging sensitivity both to social dominance^16–21^ and to events violating intuitive understanding of the physical world^22–27^, no previous research has explored the relationship between them.

Intuitive physics is one of the earliest developing ontological domains in cognition^22^. Empirical studies using behavioral (e.g., looking behaviour) and neuropsychological (e.g., brain electrical activity) measures have repeatedly shown that infants in the first year demonstrate surprise in response to physically counterintuitive events (e.g., objects suspended in mid-air with no apparent source of support), suggesting that they expect an object to follow intuitive physical principles such as gravity^22–31^. (Note that some researchers prefer to interpret the specific response (e.g., longer looking time) to physically counterintuitive events as indicative of ‘violation of expectation’ rather than of ‘surprise’ but we have adopted the latter terminology here to avoid unnecessary jargon.) Stahl and Feigenson^23^ presented 11-month-old infants with examples of objects that levitate (contrary to expectations of intuitive gravity) or teleport from one location to another without moving through space (contrary to intuitions regarding object continuity), and found that infants show preferential interest in exploring the properties of such objects. Furthermore, infants seem to apply intuitive physical principles not only in real world settings, but also in imaginary environments. Animated stimuli have been widely used in studies of infants’ cognition, partly because their superficial perceptual properties can be easily controlled. These studies show that 6-month-old infants mentally represent the occluded object^26,27^, 7- to 8-month-old infants perceive an object’s lightness in shadows by using an assumption that cast shadows dim the surface of an object^32^, 9-month-old infants apply principles of object solidity and cohesion^25^, and 12- to 16-month-old infants predict the outcome of a zero-sum conflict between two agents based on their previous spatial high- or low- positions^20^. Further, before their first birthday infants infer agents’ needs, goals and the costs of their actions in ways that take into account a wide range of physical constraints (e.g., gravity, friction, height, barriers, trenches)^25,33–35^. This extensive empirical literature demonstrates that intuitive physical principles emerge early in development, and infants expect events to follow these principles, both in physical and animated (e.g., in 3D) environments.

Studies using animated stimuli have also shown that preverbal human infants form dominance hierarchies where some systematically supplant other in zero-sum conflict^21^. For example, 6–9-month-old infants utilize number of allies, 9–13-month old infants utilize cues such as body-size, to predict which individuals will yield and prevail in the right-of-way dominance paradigm^16,17^. Twelve to 16-month old infants expect individuals in higher spatial location to gain contested resources^20^. Twelve-month-old infants expect hierarchical relationships in animated dyads to be stable over time^19^. These findings suggest that preverbal infants represent and evaluate relative social power and status based on various cues.

**Figure 1:**
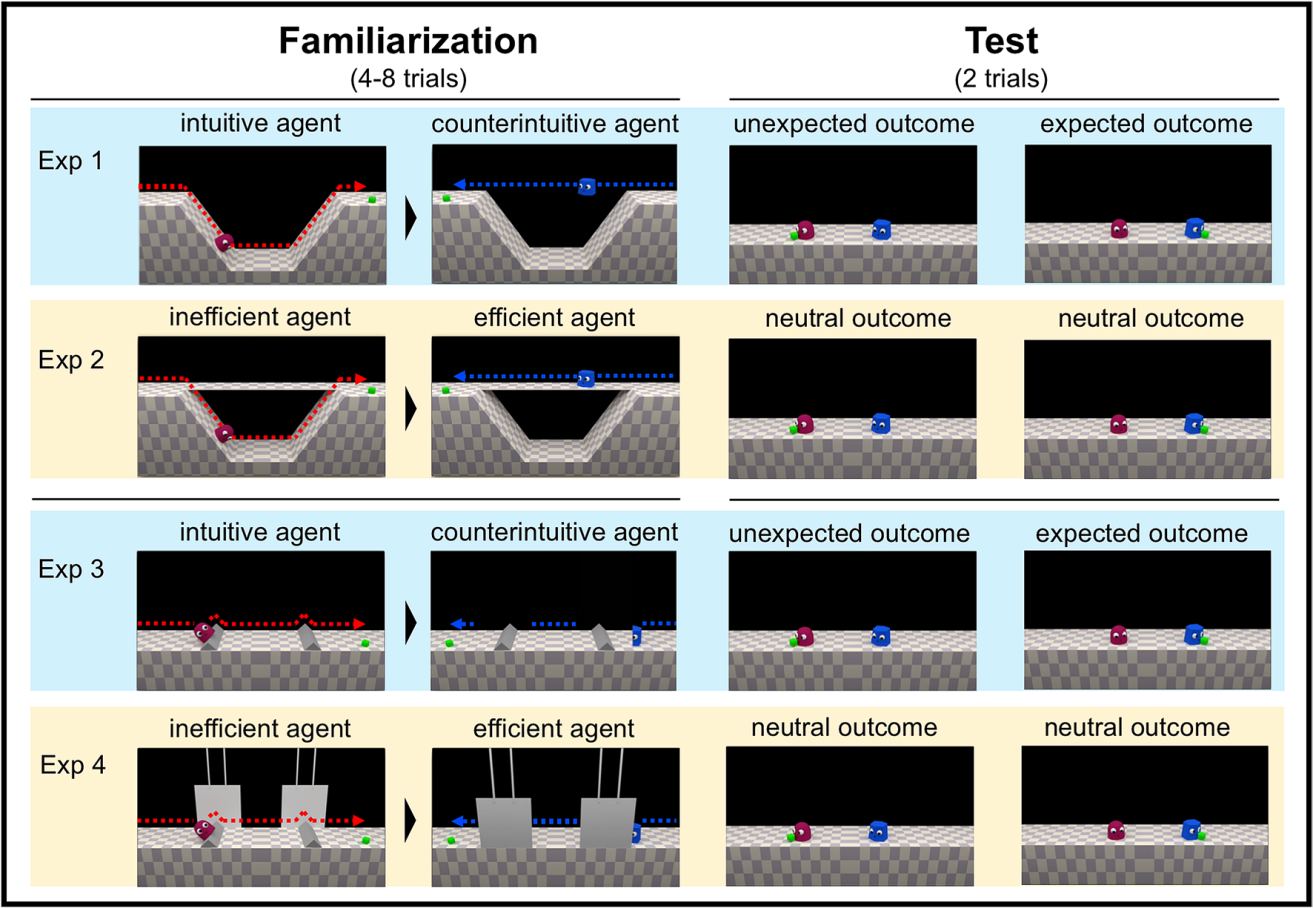
The procedure of the experiments. In the familiarization phase of Exp 1 and 3, a pair of agents gain a reward using either a physically intuitive or physically counterintuitive method. The latter was more efficient because the agents move in a straight line, which was the shortest distance to the reward (Exp 1), or disappear before each bump and quickly reappear after it, which leads faster goal-achievement (Exp 3). In Exp 2 and 4, both agents obtained the reward by moving in trajectories and speed identical to Exp 1 and 3 using physically intuitive methods (Exp 2, 4). Dotted lines present the trajectory displays of the agents (the lines do not exist in the videos), which are the same between Exp 1 and 2, and between Exp 3 and 4. In the test phase, either of the agent from the same pair of familiarization phase gains a zero-sum reward.

In the present study we consider whether physically counterintuitive capacities—capacities to achieve goals efficiently by physically counterintuitive methods—could serve as a cue for expectations of social dominance to prevail when two agents have conflicting goals^36^ (see other related studies^16,17,19,20,37,38^). In four experiments based on the violation-of-expectation paradigm, an extensively used method in infancy research that relies on looking time as an index of surprise^29,39,40^, we investigated 12- to 16-month-old infants’ looking time using animated videos in which two agents compete for a reward in a zero-sum situation (test phase)^19,20^ after the agents had retrieved a reward by overcoming an obstacle by different methods^23^ (familiarization phase; Fig. 1). Past studies have shown that young infants may regard animated geometric figures, for example, as agents with goal-directed intentions and preferences, capable of engaging in social interactions such as cooperation and interference^20,33,34,41^. Infants attributing social dominance to agents exhibiting physically counterintuitive capacities, should expect an agent who achieved goals by physically intuitive methods to withdraw and allow an agent who achieved goals efficiently by physically counterintuitive methods to obtain the reward. Accordingly, infants in the test phase should look longer at the screen (indicating surprise) after seeing a physically intuitive agent secure a reward in competition with a physically counterintuitive agent, as compared with scenarios in which the physically counterintuitive agent secures the reward in competition with an intuitive agent. Looking time following each test outcome thus served as the main dependent variable in our experimental design^16,17,19,20^. Looking times were measured as the time interval between the time point when an agent took the reward and the time point infants began to consecutively look away for 2 s or the outcome has been frozen for 60 s^20^ (see “Coding and analysis” for detailed criterion).

Experiments 1 and 3 confirmed the above prediction by showing that infants looked longer at the screen after they saw a physically intuitive agent secure a reward in competition with an agent who violated intuitive physical principles of gravity (Exp 1) or continuity (Exp3). Further control experiments (Exp 2, 4) indicated that infants’ expectations were not simply motived by the efficiency of agents in pursuing their goals, but specifically the deployment of counterintuitive capacities. Infants did not show longer looking times when a physically intuitive agent secured a reward in competition with an agent who achieved goals more effectively by physically expected methods. These findings indicate that infants expect agents exhibiting counterintuitive capacities to gain access to contested resources.

## Results

### Experiment 1

In Experiment 1 we considered a “levitation” event violating infant intuitive understanding of object supports^28,30^. In the familiarization phase, infants (*N* = 24) repeatedly watched two separate events in which each agent crossed a low valley to achieve their goal. In one event, the physically counterintuitive agent appeared to float through the air to cross a valley (thus violating the physical principle of intuitive gravity in which unsupported objects are expected to fall earthwards; see Movie S5). By contrast, in the other event, a physically intuitive agent walked down and up the same valley (see Movie S4) in order to collect the reward.

In the subsequent test phase, infants watched two video stimuli, with either one of the two agents from the familiarization phase gaining a zero-sum reward. Both agents appeared on either side of the screen, moved toward the reward, stopped, looked at each other, and then moved forward again toward each other. We introduced the action of the second forward-movement as a threatening signal to other agent, implying intention to enter into conflict. Then, after one agent again moved forward slightly, the other agent took a step backward with an averted gaze while saying “Hmmm…” in a disappointed tone. Subsequently the former agent took the object to the side it came from, stopped and lightly jumped while saying “Ahah!” in a positive tone. Then the video paused until the infants looked away from the screen for 2 s or until 60 s had elapsed from the time point in which the video paused^16,17,19,20,39^.

**Figure 2:**
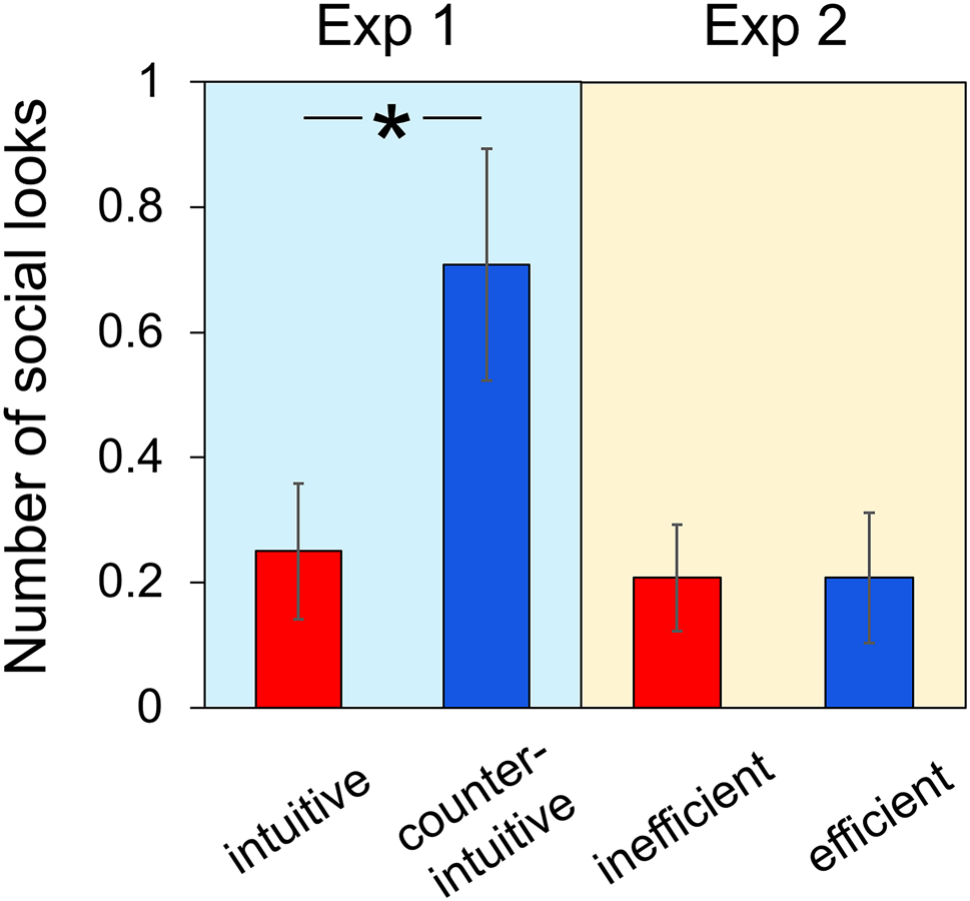
Number of social looks in the familiarization phase of Experiment 1 and 2. Intuitive = intuitive events: physically intuitive and inefficient goal-achievement events, counterintuitive = physically counterintuitive and efficient goal-achievement events, inefficient = inefficient and physically intuitive goal-achievement events, efficient = efficient and physically intuitive goal-achievement events. Error bars present the standard errors (*p < 0.05).

Results of the familiarization phase showed that more socially directed gaze—looking back to the caregiver’s face when infants were watching the events—were observed in counterintuitive trials (*M* = 0.71, *SD* = 0.91) than intuitive ones (*M* = 0.25, *SD* = 0.53; *t*_(23)_ = 2.54, *p* = 0.018, *d* = 0.519), suggesting that infants were surprised by violations of intuitive gravity (Fig. 2; visual exposure to the events was reported in SI).

**Figure 3:**
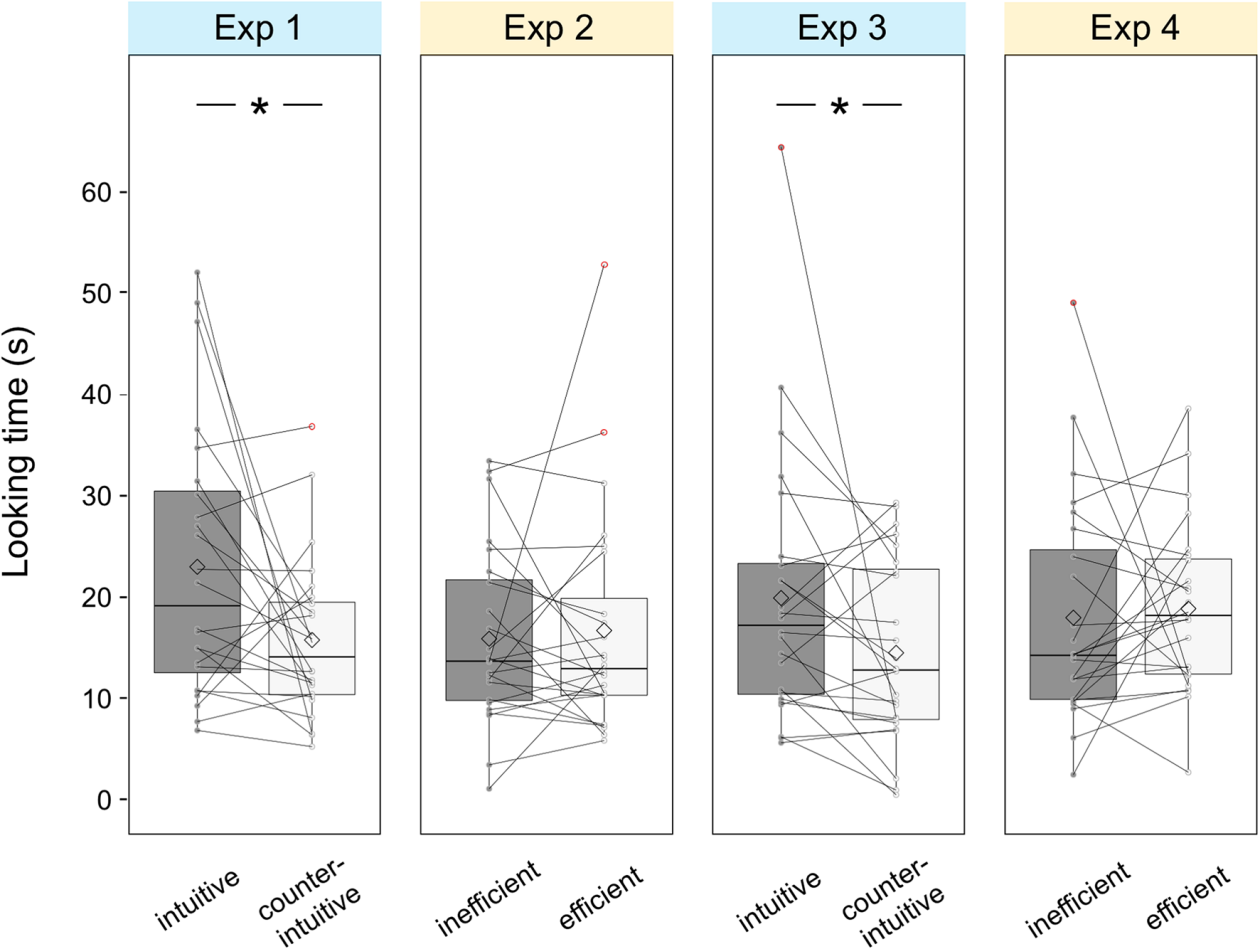
Average looking time for when either agent obtains the reward in Experiments 1–4. Diamonds indicate the means of the original looking time (not log-transformed). The horizontal line of boxes indicates upper quarterly, median and lower quarterly. Grey dots indicate looking time linked with a grey line for the same infant (within-participant). Whiskers indicate 1.5 interquartile range of upper and lower quarterly, and data that did not fit in the range was circled in red (*p < 0.05).

Results of test phase showed that infants looked significantly longer when a physically intuitive agent secured the reward (*M* = 22.95 s, *SD* = 13.41) than when a physically counterintuitive agent did so (*M* = 15.73 s, *SD* = 7.89; *t*_(23)_ = 2.33, *p* = 0.029, *d* = 0.475; Fig. 3). Post-hoc analysis tested whether social looks in the familiarization related to differentiated looking time in the test phase. We found larger looking time difference toward the two test outcomes (longer looking time on physically intuitive agent dominant tests) in infants who showed more social looks toward the counterintuitive events than the intuitive ones, compared to other infants (*t*_(22)_ = 2.57, *p* = 0.017, *d* = 1.114). Together, these findings are consistent to our hypothesis that infants expect agents achieved goals efficiently by physically counterintuitive methods to gain access to contested resources.

However, one possible explanation of the result is that infants expected the physically counterintuitive agent to obtain the reward merely because the agent proceeded more efficiently through the landscape simulated in the video (this agent moved in a straight line, which was the shortest distance to the reward) which does not require the counterintuitive property of defying gravity. To exclude this explanation, we conducted Experiment 2, a control experiment to Experiment 1.

### Experiment 2

Experiment 2 (*N* = 24) used videos depicting the same events as Experiment 1 except that there was a bridge above the valley for the agents to cross (see Movies S6, S7). In this version of the experiment, although the efficient agent moved in a straight line toward the reward (the same trajectory display with the counterintuitive agent in the Experiment 1), this agent crossed the valley via a bridge without violating infants' intuitive expectations about gravity. If the findings of Experiment 1 merely reflect an association between efficiency of the goal-achievements in the familiarization phase and the outcome of conflicts in the test phase, then we would expect infant look longer when the inefficient agent obtained the reward than when the efficient agent did so.

For the familiarization phase, in contrast to Experiment 1, number of social looks was rare during both events (*M*_inefficient event_ = 0.21, *SD* = 0.42; *M*_efficient event_ = 0.21, *SD* = 0.51), which did not differ from each other (*t*_(23)_ = 0.00, *p* = 1.000, *d* = 0.000; Fig. 2). An ANOVA on number of social looks with event type (physically counterintuitive or efficient events/physically intuitive or inefficient events) as within-subject factor and experiment (Exp 1/Exp 2) as between-subjects factors revealed a significant interaction (*F*_(1, 46)_ = 4.15, *p* = 0.047, *η*_*p*_^2^ = 0.083): infants showed more social looks during physically counterintuitive events than physically intuitive events (Exp 1; *p* = 0.018), but the number of social looks did not differ from each other when infants were watching the efficient events and the inefficient ones (Exp 2; *p* = 1.000). These suggest that infants were surprised by agents suspended in mid-air with no apparent source of support, but not by other agents moving with physical supports.

There was no significant difference between looking time for the two test trials (*M*_inefficient agent dominant_ = 15.85 s, SD = 8.76; *M*_efficient agent dominant_ = 16.67 s, *SD* = 11.14; *t*_(23)_ = 0.51, *p* = 0.613, *d* = 0.105; Fig. 3). An ANOVA on the looking time with event type (physically counterintuitive or efficient/physically intuitive or inefficient agent obtained the reward) as within-subject factor and experiment (Exp 1/Exp 2) as between-subjects factors tested the effect of the interaction (*F*_(1, 46)_ = 3.81, *p* = 0.057, *η*_*p*_^2^ = 0.077): infants tended to look longer when the physically intuitive agent obtained the reward than when the physically counterintuitive agent did so (Exp 1; *p* = 0.029), but there was no difference in looking time when both agents could be considered as physically intuitive, even though one agent was more efficient than the other (Exp 2; *p* = 0.613).

Results of Experiment 1 and 2 indicated that although infants expect agents exhibiting efficient goal-achievement by physically counterintuitive methods to prevail in zero-sum conflict over contested resources, they do not have the same expectations for agents who might be considered as merely more efficient. Furthermore, in Experiment 2, the less efficient agent should evince greater surprise given that it would be more rational for agents to take the shorter course^35^, but this had no measurable effect on expectation of social dominance on contested resources. This suggests that social dominance attribution is not due to a simple surprise without physically counterintuitive capacities.

To increase the reliability of the results so far and confirm that the previous findings were not limited to the stimuli as well as intuitive gravity, we conducted Experiment 3 using a novel context in which the physically counterintuitive agent violated the physical rule of object continuity^23,27,29^.

### Experiment 3

In Experiment 3 (*N* = 24), two bumps in the virtual landscape were inserted to serve as obstacles. To collect the reward by physically expected means, the agent needs to climb over the two bumps to reach the reward (see Movie S8), whereas the physically counterintuitive agent disappears before each bump and quickly reappears after it (the “teleportation” method; see Movie S9). The latter agent requires shorter time to obtain the reward and thus can be considered as more efficient than the former agent. As in Experiment 1, we predicted that infants expect the counterintuitive agent to outcompete the intuitive agent in securing the reward in the test phase.

During the familiarization phase, number of social looks toward caregivers were similar when infants were watching physically intuitive videos (*M* = 0.25, *SD* = 0.53) and physically counterintuitive ones (*M* = 0.08, *SD* = 0.28; *t*_(23)_ = 1.70, *p* = 0.103, *d* = 0.346). Because social looking toward the counterintuitive events rarely occurred, we did not test whether the number of social looks in the familiarization phase related to looking times in the test phase. Results of the test phase showed that looking time was significantly longer when a physically intuitive agent procured the reward (*M* = 19.87 s, *SD* = 13.40) than when a physically counterintuitive agent did so (*M* = 14.46 s, *SD* = 9.11; *t*_(23)_ = 2.46, *p* = 0.022, *d* = 0.502; Fig. 3). Again, the result is consistent to our hypothesis that infants expect agents achieved goals efficiently by physically counterintuitive methods to gain access to contested resources.

One might argue that the infants did not perceive the physically counterintuitive agent as counterintuitive, as social looks (indicating surprise) were rarely observed during the observation of the counterintuitive event. However, this might be because the duration of agents' disappearance was too short so that infants did not have enough time to look back to caregivers as they needed to follow the ongoing sequence of the video, and/or infants needed to search for the disappeared agents and thus had no time to look back. As the current data did not allow to clarify the possibilities, we designed a control experiment (Experiment 4) to test whether the physically counterintuitive property is required for the expectation of social dominance on contested resources in Experiment 3.

### Experiment 4

In Experiment 4 (*N* = 24), the physically counterintuitive event was modified so that the agents’ disappearance and re-appearance can be perceived as physically intuitive because the agents were occluded from view by two grey boards^26,27^ (see Movies S10, S11). The agent therefore still moved with trajectory displays and speed that were identical to that of Experiment 3, but would not be perceived as violating intuitive expectations about object continuity^23,27,29^. If the findings of Experiment 3 merely reflect an association between efficiency of the goal-achievements in familiarization phase and the outcome of conflicts in test phase, then we would expect infant look longer when the inefficient agent obtained the reward than when the efficient agent did so. Otherwise, infants should have no expectation on the outcome of the zero-sum conflict.

During the familiarization phase, number of social looks toward caregivers were similar when infants were watching the inefficient events (*M* = 0.21, *SD* = 0.51) and the efficient ones (*M* = 0.25, *SD* = 0.53; *t*_(23)_ = 0.33, *p* = 0.747, *d* = 0.067). In contrast to Experiment 3, results of the test phase showed that infants’ looking time did not differ after either agent secured the reward (*M*_inefficient agent dominant_ = 17.93 s, *SD* = 11.09; *M*_efficient agent dominant_ = 18.82 s, *SD* = 8.40; *t*_(23)_ = 0.73, *p* = 0.472, *d* = 0.149; Fig. 3). An ANOVA on the looking time with event type (physically counterintuitive or efficient/physically intuitive or inefficient agent obtain the reward) as within-subject factor and experiment (Exp 3/Exp 4) as between-subjects factors revealed a significant interaction (*F*_(1, 46)_ = 5.58, *p* = 0.022, *η*_*p*_^2^ = 0.108): infants looked longer when the physically intuitive agent obtained the reward than when the physically counterintuitive agent did so (Exp 3; *p* = 0.022), but there was no difference in looking time when both agents could be considered as physically intuitive (Exp 4; *p* = 0.472).

### Overall analysis of all experiments

We merged all data of four experiments and conducted a three-way ANOVA on looking times in test phase with test outcome (physically intuitive or inefficient/physically counterintuitive or efficient agent obtain the reward) as within-subject factor, and domain type (gravity/continuity) and experimental condition (manipulation/control) as between-subjects factors. The analysis revealed a significant effect of the interaction of the test outcome and experimental condition (*F*_(1, 92)_ = 9.39, *p* = 0.003, *η*_*p*_^2^ = 0.093; SI*,* Tables S2–S4). As predicted, although infants expect agents exhibiting efficient goal-achievement by physically counterintuitive methods to gain access to contested resources (Exp 1 and 3, *p* = 0.002), they do not have the same expectations for agents who might be considered as merely more efficient (Exp 2 and 4, *p* = 0.382; Analyses reported in SI indicate that removing outliers of looking time does not influence the results). These results supported our hypothesis that physically counterintuitive capacities could serve as a cue for expectations of social dominance in 12-to 16-month-old infants.

Further analysis of infants’ visual exposure to the videos in the familiarization phase shows that physically intuitive (or inefficient) events in the familiarization phase evinced longer duration of fixation in all four experiments, irrespective of experimental condition (see SI for detailed analysis). Therefore, our finding that social dominance attribution only occurs in the experimental condition but not the control condition cannot be attributed to longer perceptual exposure to the physically intuitive (or inefficient) events in familiarization.

## Discussion

The study investigated whether 12- to 16-month-old infants expect agents exhibiting counterintuitive capacities to gain access to contested resources. Infants watched animated videos in which two agents gain a reward using either a physically intuitive or physically counterintuitive method, the latter involving more efficient actions that violate constraints of gravity and object coherence and continuity that are intuitively ascribed to the ontological domain of physical objects (Exp 1 and 3). Results showed that infants looked longer when the physically intuitive agent subsequently outcompeted a physically counterintuitive agent in securing a reward. Further control experiments demonstrated that the differentiated looking behavior was absent when both agents obtained the reward by moving in trajectories and speed identical to the previous experiments but in physically intuitive ways (Exp 2 and 4). Based on the violation-of-expectation paradigm^29,39,40^, we consider the results as evidence indicating that infants expect agents exhibiting counterintuitive capacities to gain access to contested resources, and more importantly, the expectation was not simply motived by greater efficiency of the actions alone, but requires counterintuitive properties of the actions. Together, this study provides the first empirical evidence suggesting that preverbal infants link social dominance to physically counterintuitive capacities.

Past research has shown that very young infants are sensitive to and evaluating hierarchical relations among other individuals by various cues (e.g., spatial location)^16–21,38^. However, little is known by what attitude infants are evaluating the high-ranked individuals. It has been suggested that social power could be distinguished into two types: fear-based (e.g., high-ranked individuals threatening low-ranked individuals by using physical violence) and respect-based (e.g., high-ranked individuals have prestige for outstanding ability)^42–45^. Interestingly, even young children seem to distinguish between these types as “leaders” and “bullies”^46^. In the present experimental contexts, agents did not interact with each other during the familiarization process, and they did not come into physical contact with each other during the test phase (the subordinates yielded the resource to the dominant agents). Thus, we expect infants to be more likely to perceive the agents exhibiting physically counterintuitive capacities as respect-based target than as fear-based target^20,37,47^.

As the lack of the physical contact, one may argue that it is not clear whether infants saw the test events as competitive interactions or as cooperative interactions (an act of kindness from the agent who let another agent to take the resource). Although it is hard to perfectly distinguish between these accounts by experimental manipulations because it is difficult to establish precisely how infants are evaluating the interaction of the agents. However, note that in our stimuli, both the agents moved toward the reward, stopped, looked at each other, and then approached each other again^20^. We introduced the second approach to indicate both agents’ willingness to engage in conflict, which is obviously different from the form of interactions in which a kind helper intends to help someone to achieve a goal^48^. Therefore, even though the subordinate finally yields, we think that it is difficult to interpret this as an act of kindness. However, further studies may explore whether it is theoretically reasonable to predict that people expect intuitive agents to be “more kind” than counterintuitive agents.

Results of social looks in Experiment 1 and 2 suggested that infants were surprised by agents violating expectations of intuitive gravity, but not by other agents moving with physical supports. Moreover, the contrasting results of the experimental condition (Exp 1, 3) and the control condition (Exp 2, 4) also indicated that expectations of social dominance were not simply motived by greater efficiency of the actions alone, but by the counterintuitive properties of the actions. At first glance, there seems to be a possible alternative explanation that infants were surprised to discover that an agent has enough internal force to hover in mid-air, like a bird, without assuming that this agent violates physical principles. However, the claim that levitation/teleportation are results of “agents’ internal power” is unconvincing. What is “internal power” in this context, if not the counterintuitive ability to violate expectations of intuitive physics? Assumption of any kinds of ‘internal power’ should be driven by the deviation between the observed phenomenon and the intuitive calculation about the phenomenon based on knowledge and beliefs. While birds and planes may be commonly encountered and unsurprising, that does not mean that they are not also counterintuitive in the strict sense of violating intuitive expectations in the physical ontological domain according to which unsupported objects should move earthwards and objects should move in continuous paths.

To reduce the influence of the caregivers on infants’ behavior, we instructed caregivers not to interact with infants during the experiment. We did not instruct caregivers to close their eyes or to wear a pair of opaque glasses for the duration of the study. We reasoned that being able to observe what is happening during the experiment would be reassuring to caregivers. In previous tests, when caregivers had been asked to close their eyes to reduce experimental noise, most nevertheless continued to keep their eyes open^20^. We further reasoned that allowing caregivers to keep their eyes open might elicit higher rates of social looking in the familiarization phase^49^. We acknowledge the risk, however, that infants’ looking behavior was influenced by caregivers’ conscious/unconscious response towards the stimuli. Future studies should test whether caregivers’ knowledge of the stimuli affects infants’ behavior in intuition–violation studies on early social cognition.

The present study tested whether observing efficiently goal-directed events that violate intuitive gravity and continuity trigger expectations of social dominance. This investigation was prompted by previous research suggesting that young infants have early emerging intuitive expectations in the domain of physics^22–24,27–31^, that violations of these core expectations serve as a fundamental feature of supernatural agent constructs, and that supernatural agents are sometimes ascribed positions of authority^13–15^. Previous research with adults has shown that some supernatural constructs, for example involving simple breaches of intuitive expectations in a particular domain (e.g. person who levitates) are less cognitively taxing than others, for example involving transfers across ontological domains (e.g., the transformation of a frog into a stone), making the latter more surprising^50^. Such constructs should also be mobilized in studies with infants to see if they are capable of driving even stronger expectations on social dominance. Moreover, our study focuses only on violations of intuitive physics, whereas violations of other intuitive domains such as psychology (e.g., “Omniscient of a God”) or biology (e.g., “Immortality”) are also common motifs in mythical stories and religions^13–15,51^, and worthy of further exploration in studies of early reasoning about supernatural agents. For instance, one may want to test whether infants attribute social dominance to an agent who falsely believes a toy was in a wrong location but can always find the toy^52^. Finally, in order to understand the psychological mechanisms linking counterintuitive qualities to expectations of social dominance, future research should test a broader range of experimental manipulations, for example by exploring whether a merely counterintuitive display, without (efficient) goal-achievements, similarly elicits expectations of social dominance. The current study did not test the simply effect of counterintuitive properties because we think that it is hardly to theoretically build a general argument about the relationship between counterintuition and social dominance without considering the meaning of counterintuitive properties to goal-achievements. Counterintuitive agents would not always seen as more powerful than agents who do not violate intuitive principles (e.g., a mind trapped in a statue is counter-intuitive, whereas we would not expect it to be more powerful over an entity controlling a moving body). We predict that counterintuitive properties can be seen as a source of social power only insofar as they make agents more likely to achieve their goals. The possibly is worth further investigations.

In addition, there is considerable scope for future developmental studies to explore a wider range of expectations prompted by counterintuitive attributes. For example, do infants expect agents exhibiting counterintuitive capacities to be, not only socially dominant in zero-sum conflict over contested resources, but also more knowledgeable, trustworthy, competent, or prosocial? It is important to investigate these possible outcome variables because, although there is considerable evidence from anthropology that humans possessing (seemingly) supernatural powers are commonly expected to exhibit a range of social skills distinguishing them from lesser mortals, little is known about the developmental origins of these expectations.

The connection between supernatural power and authority has been observed universally and throughout history^1–8^. Our finding that possession of physically counterintuitive capacities motivate expectations of social dominance at such an early stage of development suggests that this is a fundamental feature of human nature. This does not necessarily mean that the link between supernatural power and social dominance is an evolved adaptation, given that the religious thought itself may plausibly be a multiple by-product of the normal operation of human cognition^53,54^. Nevertheless, infants’ evaluation bias observed in the current study would not only help to explain the ubiquity of the link between supernatural agency and authority in human societies over the ages but would also suggest that this link may be difficult to eradicate in the longer run despite the rise of secularism in some regions of the world in recent history.

## Methods

### Participants

Each of the four experiments included a final sample of 24 12- to 16-months old infants (see Participants in SI for the power analysis and data exclusion). The mean age of participants was 14 months and 4 days in Experiment 1 (SD = 43.92 days; 15 girls and 9 boys), 14 months and 5 days in Experiment 2 (SD = 43.50 days, 13 girls and 11 boys), 14 months and 24 days in Experiment 3 (SD = 45.20 days, 13 girls and 11 boys), and 15 months and 1 days in Experiment 4 (SD = 48.1 days; 14 girls and 10 boys). Written consent was obtained from all caregivers before the experiment. All participants were recruited and tested at the BabyLab in Kyushu University Hospital. The study was approved by the ethical committee of Kyushu University (2017-012), and was conducted in accordance with the Declaration of Helsinki.

### Set-up

All experiments were conducted in a partitioned space in a quiet room (SI, Set-up). Infants were seated on the lap of the caregivers who were seated on a floor pillow, 145 cm away from a 55-in. television on which the visual and audio stimuli were presented^20^. Four video cameras recorded the experiment from the different angles. Outside the booth, two experimenters controlled the stimuli presentation and undertook online coding. Role-sharing ensured that the experimenters were blind to the experimental conditions: the experimenter who controlled or coded the test phase did not possess prior knowledge about the agents’ conditions (e.g., physically counterintuitive).

### Stimuli and procedure

The experiments used 3D animated video stimuli created in Blender^55^ (2.79b, Blender Foundation; https://www.blender.org/), synchronized with custom audio tracks in Final Cut Pro X (Version 10.2, Apple Inc.; https://www.apple.com/jp/final-cut-pro/). 3D videos created by Blender or other game engines have previously been used in studies of infant studies including the studies to test infant’s intuitive understanding of the physical world^20,33,56^. Stimuli were presented to participants as animation movies of agents (geometrical figure with two eyes and on nose). One pair included a red agent and a blue agent; the other included an orange agent and a green agent. All of the agents were of the same size (e.g., the height and width of the cube agent was 7.5 cm and 9 cm). Each infant watched animated videos featuring one of these pairs during familiarization and test phases; pairs, as well as the identities of the agents, were counterbalanced between subjects (SI, Stimuli pattern).

The movie of all experiments mainly consists of three parts: the warm-up phase, the familiarization phase and the test phase (SI, Design). The warm-up phase was identical in all experiments, and aimed to familiarize infants to the competitive context of the test phase (e.g., video freezes after one of the agents has obtained the reward; SI, Stimuli and procedure).

In the familiarization phase, the infant watched two agents obtain the reward by using different methods (see Fig. 1, SI, Stimuli and procedure). During each trial of the familiarization phase, a cube-shaped reward fell down on one side of the stage. Then one of the paired agents appeared from the other side of the stage. The agent then began to move while overcoming a physical obstacle (i.e., crossed a valley), collected the reward, went back, and then exited the screen from the side it appeared. The sides on which the object/agent appeared were counterbalanced (SI, Stimuli pattern). The events in the familiarization phase differed by experiments (see “Results”).

To ensure that infants had sufficient exposure to the events to understand that the agents had the goal of collecting the reward by physically intuitive/counterintuitive methods, we applied the stimuli presentation scheme based on previous studies^16,17,46^. Specifically, each event repeated for a minimum of two trials and a maximum of four trials. Because there would be individual differences regarding whether and when to lose interest in the familiarization events, the end of the familiarization depended on whether the infant looked away from the screen: the events were repeated twice for each agent, then further repeated until the infant either looked away from the screen for 2 s or watched each video 4 times.

The sequence was fixed, beginning with the physically intuitive events (or the corresponding events in control experiments, e.g., inefficient events). This was to enhance the violation of intuition: presenting the intuitive event in the first trial was intended to habituate infants to expect that agents should move according to physical principles (e.g., with physical support).

For the test phase we followed previous infant’s studies testing infants’ expectation of hierarchical relationships^19,20,36^. In all four experiments, the test trials were identical (see Movie S12). Each infant watched two test videos (counterbalanced; SI, Stimuli pattern) in which either one agent retrieved the reward, and the looking time was measured from the moment at which an agent obtained the reward.

### Coding and analysis

The familiarization phase and the test phase were coded both online and offline to measure if infants looked away for 2 consecutive seconds from the screen (SI, Coding). The purpose of online coding was to manage the timing of experimental procedure. The coding was conducted independently by two different experimenters to ensure that they were blind to the experimental conditions. To measure the exact looking time for test trials, the video recordings of the experiments were coded offline again. Looking times during the test event were measured as the time interval between the moment the agent took the reward (before the screen had paused) and the moment infants began to consecutively look away for 2 s or 60 s had elapsed from the time point when the screen had frozen. The maximum possible looking time was 64.35 s. Each video was coded frame-by-frame whether or not infants were looking at the screen. One coder coded all trials and another coder independently coded 50% of the trials. The interclass correlation of coders was 0.973 (95% CI 0.960–0.982, *p* < 0.001).

Looking time was log-transformed for data analysis^57^. We conducted two-tailed t-test to test whether a significant difference in looking time can be observed between two test trials (either of the agents obtained the reward). Furthermore, we did not have (theoretical) hypothesis regarding the relationship between the order of the test trials/sex of participants and infants’ looking time during the test phase. We conducted exploratory analyses with analysis of variance (ANOVA) to confirm that these two factors did not influence the looking time (for results of the ANOVAs, see SI)^17^.

To confirm that infants were surprised more by the physically counterintuitive events than by the physically intuitive events in the familiarization phase, we investigated whether infants looked back to the caregiver’s face in more trials when they were watching physically counterintuitive events than physically intuitive events. Social looking has been proposed as a robust method of measuring surprise at violation of expectations^49,58^. We used social looking because looking time was not appropriate to measure surprise for the current familiarization design (see SI for detailed explanations). Social looking was coded to measure whether infants looked back to bring the caregiver’s face into focal view in each familiarization trial^49,58^. Two observers who were unaware of the aim of the study coded all the trials. The inter-observer agreement was 0.968 (95% CI 0.953–0.982; Gwet's AC)^59^.

## Supplementary Information

Supplementary Information 1.

Supplementary Video 1.

Supplementary Video 2.

Supplementary Video 3.

Supplementary Video 4.

Supplementary Video 5.

Supplementary Video 6.

Supplementary Video 7.

Supplementary Video 8.

Supplementary Video 9.

Supplementary Video 10.

Supplementary Video 11.

Supplementary Video 12.

Supplementary Information 2.

Supplementary Information 3.

## Data Availability

The dataset is available as Supplementary Dataset.
