# Supplementary material for: Preverbal infants expect agents exhibiting counterintuitive capacities to gain access to contested resources
Source: Sci Rep. 2021 May 25;11:10884. doi: 10.1038/s41598-021-89821-0 (PMC8149634; doi:10.1038/s41598-021-89821-0)
Supplement: Supplementary file 1 — Supplementary Information 1. [file 41598_2021_89821_MOESM1_ESM.docx]

Titles and Captions of Movie Files for

**Preverbal infants expect agents exhibiting counterintuitive capacities to gain access to contested resources**

Xianwei Meng^1,2,3*†^, Yo Nakawake ^1,4,5*†^, Kazuhide Hashiya^1^, Emily Burdett^5,6,7^,

Jonathan Jong^5,6^, and Harvey Whitehouse^5*^

^1^ Faculty of Human-Environment Studies, Kyushu University

^2^ Graduate School of Human Sciences, Osaka University

^3^ Center for Baby Science, Doshisha University

^4^ School of Economics and Management, Kochi University of Technology

^5^ Centre for the Study of Social Cohesion, University of Oxford

^6^ Belief, Brain and Behaviour, Coventry University

^7^ School of Psychology, University of Nottingham

†These authors contributed equally.

*Correspondence concerning this article should be addressed to Xianwei Meng (mokeni1211@gmail.com), Yo Nakawake (yo.nakawake@anthro.ox.ac.uk), and Harvey Whitehouse (harvey.whitehouse@anthro.ox.ac.uk).

**Movie S1.** The calibration stimulus

**Movie S2.** The stimuli of the warm-up phase

**Movie S3.** The introduction of the familiarization

**Movie S4.** Experiment 1, the familiarization stimulus of the physically intuitive agent

**Movie S5.** Experiment 1, the familiarization stimulus of the physically counterintuitive agent

**Movie S6.** Experiment 2, the familiarization stimulus of the inefficient agent

**Movie S7.** Experiment 2, the familiarization stimulus of the efficient agent

**Movie S8.** Experiment 3, the familiarization stimulus of the physically intuitive agent

**Movie S9.** Experiment 3, the familiarization stimulus of the physically counterintuitive agent

**Movie S10.** Experiment 4, the familiarization stimulus of the inefficient agent

**Movie S11.** Experiment 4, the familiarization stimulus of the efficient agent

**Movie S12.** Sample of the stimuli in test trials
