# Supplementary material for: Preverbal infants expect agents exhibiting counterintuitive capacities to gain access to contested resources
Source: Sci Rep. 2021 May 25;11:10884. doi: 10.1038/s41598-021-89821-0 (PMC8149634; doi:10.1038/s41598-021-89821-0)
Supplement: Supplementary file 15 — Supplementary Information 3. [file 41598_2021_89821_MOESM15_ESM.pdf]

Supplementary Information for

Preverbal infants expect agents exhibiting counterintuitive capacities to gain access to contested resources

Xianwei Meng[1,2,3*†], Yo Nakawake [1,4,5*†], Kazuhide Hashiya[1], Emily Burdett[5,6,7], Jonathan Jong[5,6], and Harvey Whitehouse[5*]

[1] Faculty of Human-Environment Studies, Kyushu University

[2] Graduate School of Human Sciences, Osaka University

[3] Center for Baby Science, Doshisha University

[4] School of Economics and Management, Kochi University of Technology

[5] Centre for the Study of Social Cohesion, University of Oxford

[6] Belief, Brain and Behaviour, Coventry University

[7] School of Psychology, University of Nottingham

†These authors contributed equally.

*Correspondence concerning this article should be addressed to Xianwei Meng (mokeni1211@gmail.com), Yo Nakawake (yo.nakawake@anthro.ox.ac.uk), and Harvey Whitehouse (harvey.whitehouse@anthro.ox.ac.uk).

This PDF file includes:

- Stimuli pattern
- Participants
- Set-up
- Design
- Stimuli and procedure
- Coding
- Infants' visual exposure to the videos in the familiarization phase
- Overall analysis of all experiments (on looking time of the test phase)
- Exploratory analyses of the test phase
- References

Stimuli pattern

Pattern	Warm-up*	Experiment 1 & 2 (Support/Gravity)			
			the left side	the right side	
			(*From the participant's view)		
1	B	dominant in 1st test	C/E	I/I	
2	D		C/E	I/I	dominant in 1st test
3	A	dominant in 1st test	I/I	C/E	
4	C		I/I	C/E	dominant in 1st test
5	C		C/E	I/I	dominant in 1st test
6	A	dominant in 1st test	C/E	I/I	
7	C		I/I	C/E	dominant in 1st test
8	C	dominant in 1st test	I/I	C/E	
9	A	dominant in 1st test	C/E	I/I	
10	B		C/E	I/I	dominant in 1st test
11	B	dominant in 1st test	I/I	C/E	
12	D		I/I	C/E	dominant in 1st test
13	B		C/E	I/I	dominant in 1st test
14	D	dominant in 1st test	C/E	I/I	
15	D		I/I	C/E	dominant in 1st test
16	A	dominant in 1st test	I/I	C/E	

C/E = Counterintuitive/Efficient agent
I/I = Intuitive/Inefficient agent

Pattern	Warm-up*	Experiment 3 & 4 (Continuity)			
			the left side	the right side	
			(*From the participant's view)		
1	B	dominant in 1st test	C/E	I/I	
2	D		C/E	I/I	dominant in 1st test
3	A	dominant in 1st test	I/I	C/E	
4	C		I/I	C/E	dominant in 1st test
5	C		C/E	I/I	dominant in 1st test
6	A	dominant in 1st test	C/E	I/I	
7	C		I/I	C/E	dominant in 1st test
8	C	dominant in 1st test	I/I	C/E	
9	A	dominant in 1st test	I/I	C/E	
10	B		I/I	C/E	dominant in 1st test
11	B	dominant in 1st test	C/E	I/I	
12	D		C/E	I/I	dominant in 1st test
13	B		I/I	C/E	dominant in 1st test
14	D	dominant in 1st test	I/I	C/E	
15	D		C/E	I/I	dominant in 1st test
16	A	dominant in 1st test	C/E	I/I	

C/E Counterintuitive/Efficient agent
I/I = Intuitive/Inefficient agent

*The pattern of warm-up phase
A = The bigger agent appeared in the first trial, from the left side of the screen.
B = The smaller agent appeared in the first trial, from the right side of the screen.
C = The bigger agent appeared in the first trial, from the right side of the screen.
D = The smaller agent appeared in the first trial, from the left side of the screen.

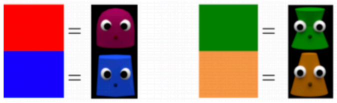

Fig. S1. Stimuli pattern.

Participants

The final sample included ninety-six 12-16 month old Japanese infants (i.e., n = 24 for each experiment). The sample size was determined based on a priori power analyses taking into account the results of previous infant studies on social dominance (especially, Pun, Birch & Baron, 2016) [1–4]. Specifically, a minimum of 24 participants in each experiment was required to detect potential effects using three-way repeated-measures analysis of variance (ANOVA) with effect size between the medium and large level: partial $\eta^2 = .10$, $\alpha = 0.05$, and $1\text{-}\beta = 0.80$ [5]. Written consent was obtained from all caregivers before the experiment. All participants were recruited and tested at the BabyLab in Kyushu University Hospital. The study was approved by the ethical committee of Kyushu University (2017-012), and was conducted in accordance with the Declaration of Helsinki. Below is detailed information about the participants in each experiment.

Experiment 1

Twenty-four infants participated (15 girls and 9 boys; M_{age} = 423.9 days, *SD* = 43.92, range = 351-496). Eleven additional infants were excluded because they did not direct their attention to the animation (*n* = 7), because they did not watch the critical event in which one agent took the reward and thus missed the onset of coding in test phase (*n* = 3), or because the coder accidentally ended the test before the infant looked away from the screen for 2 seconds (*n* = 1).

Experiment 2

Twenty-four infants participated (13 girls and 11 boys; M_{age} = 424.6 days, *SD* = 43.50, range = 370-496). Eleven additional infants were excluded because they were fussy and did not direct their attention to the animation (*n* = 5), because their eyes were out of the camera so that coding was impossible (*n* = 3), or because the caregivers adjusted the infants' positions while they were watching the test video which made it difficult to judge whether infant faced the screen due to their own will or parental intervention (*n* = 3).

Experiment 3

Twenty-four infants participated (13 girls and 11 boys; M_{age} = 443.8 days, *SD* = 45.20, range = 366-516). Seven additional infants were excluded because they did not direct their attention to the animation (*n* = 3), because the caregivers interfered with the infants' looking behavior toward the screen in test phase by making eye contact or adjusting infants' position (*n* = 3), or because the infant's sibling talked outside the experiment booth and thus interfered the infant's attention towards the videos (*n* = 1).

Experiment 4

Twenty-four infants participated (14 girls and 10 boys; M_{age} = 450.8 days, *SD* = 48.11, range = 372-513). Nine additional infants were excluded because they did not direct their attention to the animation (*n* = 2), because their eyes their eyes were outside of the viewing range of the camera so that coding was impossible (*n* = 2), because they did not watch the critical event in which one agent took the reward and thus missed the

onset of coding in test phase (*n* = 2), because their attention was drawn by the voice of the sibling outside the booth (*n* = 1) because they were paying attention to something else (e.g., their shoes) (*n* = 1), or because of an experimental error regarding video presentation order (*n* = 1).

Set-up

The same testing materials was used across four experiments. The experiments were conducted in a partitioned space (w×d×h = 195×315×150 cm) in a quiet room at the BabyLab. Infants sat on their caregivers' laps, approximately 145 cm from a 55-inch (121.54×68.45 cm) SONY BRAVIA X8500E television on which the experimental visual and audio stimuli were presented. To enhance the reality of the experimental stimuli (e.g. to enhance the sense that the agent was actually walking on the ground, given that the physically intuitive agent should not be perceived as flying or levitating), we set the display on the carpeted floor so that the bottom edge of the display was directly adjacent to the floor, and we also asked caregivers to sit on the floor. Four video cameras recorded the experiment; three hidden cameras captured whether infants were looking at the screen from the top, right, and left sides of the television, one camera captured the stimuli from the back of the participants. The recordings were later synchronized using a video mixer (Roland V4-EX). Outside the booth, two experimenters controlled the stimuli presentation using Microsoft PowerPoint 16.16 and coded lively infants' looking time data online through a video screen (23-inch TFT, 1920×1080 pixels) using software coded by Visual Basic (not used for off-line coding of the test phase; https://github.com/YNakawake/timewatcher_socdominance).

Design

All four experiments were within subject designs involving a violation-of-expectation paradigm. Experiment 1 and 3 aimed to test the hypothesis that infants mentally represent a physically counterintuitive agent as socially dominant. In the test phase of Experiment 1 and 3, infants were presented with both an Expectation-consistent test outcome (the physically counterintuitive agent outcompetes a physically intuitive agent in securing a reward) and an Expectation-violated test outcome (the physically

intuitive agent outcompetes a physically counterintuitive agent in securing a reward). The dependent variable was the length of looking time at each test outcome. The independent variable was the category of the test outcomes (expectation-consistent or expectation-violated). Experiment 2 and 4 were control experiments of 1 and 3 respectively, to exclude alternative explanations of the results. In the test phase of Experiment 2 and 4, infants were presented video stimuli that were identical to that in Experiment 1 and 3, whereas neither of the test outcomes could be considered as expectation-consistent or expectation-violated, given that both agents appeared in these tests were physically intuitive, as detailed below (see also *Fig. 1* in the main article).

Stimuli and procedure

Caregivers were briefed and informed consent was obtained outside of the experimental booth prior to the study. Entering the experimental booth, caregivers were instructed not to interfere with infants by uttering, pointing, or making eye contact if infants tried to engage the caregiver during the experiment. Infants were seated on the lap of the caregivers who were seated on a floor pillow, 145 cm away from the screen. During all experiments, it was dimly lit inside the booth. After a pre-experimental calibration phase, the video stimuli were presented. The experiments each consisted of three phases: the warm-up phase, familiarization phase (4-8 trials) and the test phase (2 trials). In each phase, the corresponding type of video stimuli was presented. Each experiment lasted approximately 6 minutes.

Calibration

The calibration phase aimed to create referential indexes of infant gazing patterns to enable the coders to judge whether or not infants were watching the screen during offline coding. Specifically, we recorded the scenes in which each infant was looking at each end of the screen, and used them as the individual based referential index. The calibration phase comprised a white screen with six yellow diamond-shaped geometric figures which sequentially appeared on each ends of the screen, and spun around accompanied by a chiming sound to get the attention of infants (See *Movie S1*).

Warm-up phase

The warm-up phase was included to familiarize infants to the competitive context of the test phase. The videos used in this phase were based on Thomsen et al.'s (2011) study, which demonstrated that 10-month-old human infants use body size as a cue to infer social dominance in competitive context [1].

We included the warm-up phase to habituate infants to the paradigm of the test phase for a few reasons. First, we wanted to reduce infants' confusion after they saw the test videos pause at the end of each trial, during which time we recorded their gazing time. In a pilot study, we presented similar experimental stimuli four times to infants without the warm-up phase. We found that infants frequently looked alternately to the caregivers and the screen when the test video paused in the initial trial. But, these behaviors were scarcely observed in the following three tests. Therefore, infants probably felt confused when the video stopped for the first time. Such response may lead to larger variance in infants' looking time across trials, and reduce the power of detecting the possible effects of experimental manipulation. Thus, to avoid these responses, we inserted a warm-up-phase to habituate infants that the screen will stop after viewing the competition. Second, we wanted to familiarize infants to the competitive context of the test, and that the socially dominant agent should obtain the reward in this context. Specifically, two agents in obviously large and small sizes appeared and competed for a reward.

The warm-up phase consisted of the three parts (see *Movie S2*). In the first, infants watched a video in which a small (7.5 cm in diameter) and a big (13 cm in diameter) spherical agent appeared in the center of the screen, and then moved in the opposite direction and exited the screen while saying "Hm" in a negative tone. The agents different only in body size: each had a dark green spherical body with two eyes and a nose.

In the second part, infants were presented with videos that displayed agents collecting a reward. Videos started with a yellow cube-shaped reward falling down on one side of the stage. Then either the small or big agent appeared from the other side of the stage, collected the reward, then went back and exited the screen from the side it appeared. Then the other agent repeated the same event. The starting location and order

of the agent (whether the big or the small agent appeared the first) were counterbalanced (see *Fig. S1*).

The final part consists of two warm-up test trials almost identical to the actual test phase. Here, infants watched both the small and big agent compete for the reward: the big agent always outcompeted the small agent by obtaining the reward. Videos started with a yellow cube-shaped reward falling into the middle of the screen. Then the two agents appeared together simultaneously from the either side of the screen. Both agents approached the reward at the same speed. After approaching, both agents stopped and gazed at each other, and again they slowly moved forward as though cautious of one another, before stopping again. Then, the big agent moved forward slightly, whereas the small agent took a step backward with an averted gaze while saying “Hmmm...” in a disappointed tone. Subsequently the big agent took the object back to the side it came from, stopped and lightly jumped while saying “Ahaha!” in a positive tone. Then the animation paused for 5 seconds before ending. This trial was repeated twice (with the big agent obtaining the rewards both times), and the sides that agents appeared were counterbalanced (for the counterbalance information, see *Fig. S1*). The length of the entire warm-up phase was 83 seconds.

Familiarization phase

Following the warm-up phase, the familiarization phase began. In the familiarization phase, the infant watched two agents obtain the reward by using different methods. Two pairs of agents, similar to those in the warm-up phase featured in these events.

In Experiment 1 and 3, we manipulated the way in which the agents overcame the obstacles: one agent—the physically intuitive agent—collected in a way that did not violate intuitive expectations (see below), while the other—the physically counterintuitive agent—overcame the obstacles in ways that violated intuitive physical expectations (see below). For each infant, the physically intuitive and physically counterintuitive agents always differed from each other in color, but the colors of the agents always remained the same across presentations of the events for each infant (red vs. blue or orange vs. green). The events were presented a minimum of 2 times and

maximum of 4 times each: from the third presentation, the familiarization was terminated when infants looked away from the screen for >2 s, or when infants had watched each event four times in total (See *Stimuli and procedure* in the main article).

Experiment 1 and 3 differed in the type of intuitive physical expectation that was violated in order to overcome an obstacle. In Experiment 1, the physically counterintuitive agent violated infants' intuitive expectations about gravity. Specifically, agents were shown crossing a valley. The physically counterintuitive agent crossed the valley by going straight over the valley without physical support (see *Movie S5*), as though there were an invisible bridge over the valley (the length of trajectory was 121.5 cm; the average speed of the movement was 0.055 m/s), whereas the physically intuitive agent crossed the valley by climbing down and up the valley (the length of trajectory was 150.5 cm; the average speed of the movement was 0.068 m/s; see *Movie S4*). Although the two agents overcame the valley via different paths, the duration time was the same (22 s). In Experiment 3, the physically counterintuitive agent violated infants' intuitive expectations about object continuity. Specifically, agents were shown overcoming bumps blocking their path (the length of trajectory was 126.5 cm). The physically counterintuitive agent disappeared just before the hill and re-appeared at the place beyond the bump (see *Movie S9*), whereas the physically intuitive agent walked up and down the bump (see *Movie S8*). To avoid infants looking away from the screen during the disappearance, the duration of the physically counterintuitive agent's disappearance (the average speed of the movement was 0.082 m/s) was set to be shorter than that of the physically intuitive agent's movements (the average speed of the movement was 0.063 m/s).

Experiments 2 and 4 were control experiments of 1 and 3, respectively. In Experiment 1, the physically counterintuitive agents' path may be seen as more efficient than the physically intuitive agents', because it moved in a straight line. Thus in Experiment 2, we replaced the physically counterintuitive event with one in which the agent crossed the valley via a bridge: this involved moving in a straight line without violating infants' intuitive expectations about gravity (see *Movie S7*; see also *Movie S6* for the inefficient event).

In Experiment 3, the movement of the physically counterintuitive agent was faster than the physically intuitive agents. Thus, in Experiment 4, the physically counterintuitive event was modified so that the agent's disappearance and re-appearance was occluded from view by two grey boards placed specifically for this purpose: the agent therefore still moved more quickly than the physically intuitive agent, but without necessarily violating intuitive expectations about object permanence (see *Movie S11*; see also *Movie S10* for the inefficient event).

The experimental manipulation aimed to test whether counterintuitive behaviour affects expectations of social dominance. Therefore, any difference on low-level visual characteristics of the agents' movements (e.g., speed) between the conditions – factors we were not focusing on but which could affect the attribution of social dominance – should be eliminated. That is, agents in both experimental and control conditions (e.g., intuitive agents in Exp1 and Exp 2) should advance at the same rate. To achieve this, we ensured that agents exhibited the same visual characteristics in terms of trajectories and speed across conditions. We also used different contexts (i.e., different stages in the animation) to manipulate counterintuition of the movements. These manipulations resulted in several points regarding agents' movements which might be perceived as slightly "anomalous". Specifically, the counterintuitive/efficient agents in Exp 1 and 2 slightly moved sideward towards the background before crossing the valley. This manipulation was applied to ensure that we could remove/insert a bridge above the valley without modifying agents' movement trajectories cross conditions. Furthermore, the counterintuitive/efficient agents in Exp 3 and 4 disappears not all at once but by sliding behind an invisible/visible occluding object. This manipulation was applied to ensure that we could remove/insert the boards without modifying agents' movement trajectories. It could be argued that these manipulations made the unexpected movements less like levitation or teleportation. However, we believe that they were required to achieve the intended experimental design allowing us to test the main hypothesis while avoiding other factors we were not focusing on. More importantly, measures of social looks suggested that infants were surprised by agents suspended in mid-air with no apparent source of support, but not by other agents moving with physical support (see *Results* in the main article), which showed that the

stimuli we adopted succeeded in manipulating infants' evaluations of the events in terms of intuitive/counterintuitive properties.

Test phase

The animation was identical to that in the last two trials of the warm-up test, except the following: First, instead of the big and small agents, infants watched the agents from the previous familiarization phase. Second, each trial ended if infants looked away from the screen for >2 s, or after 60 s elapsed from the time point the screen paused.

Coding

All the sessions were both coded online and offline. The entire experiment was recorded with four video cameras. Online coding was conducted to manage the timing of experimental procedure. The coders measured if infants looked away for consecutive 2s, which is the criterion for terminating the current trial and proceed to the next trial. In the familiarization phase, this coding was applied from the fifth trial. In the test phase, this coding was applied to both test trials. Two coders measured the time by pressing a key while infants were looking away from the screen with an aid of the computer programme built on Visual Basic (https://github.com/YNakawake/timewatcher_socdominance). The familiarization and the test trials were coded by different coders. Each coder only watched their part, thus the coder of the test phase was blind to the events in the familiarization phase. In case of ambiguity (e.g. not clear whether infants were looking at the screen or were looking away), the coder was instructed to consider it as infants were looking at the screen; thus the coder reset (stopped pressing the key) and waited for another extra 2s to terminate and proceed to the next step.

In the calibration phase, we created an individual based referential index to establish a method of coding instances in which infants were looking at the screen versus looking away. Using this referential index in the test phase, precise looking time was coded offline by measuring frame-by-frame whether or not infants were looking at the screen. One coder coded the whole recorded test trials and another coder independently recoded 50% of the data. The test was coded separately from the familiarization, thus coders were blind to the hypothesis and events of familiarization. Looking time at the test

event were measured as the time interval between the moment the agent took the reward and the moment infants began to consecutively look away for 2s or 60s had elapsed from the time point when the screen had paused. The interclass correlation of coders was 0.973 [CI (0.960, 0.982), $P < 0.001$]. No trials were terminated before the cutoff point coded offline, except one trial. We excluded the data of this participant from data analyses due to an operation error of the experimenter ($n = 1$, Experiment 1).

Based on this coding scheme, we checked if the participants met two exclusion criteria. Firstly, we included the data of participants whose visual exposure to the videos (i.e., total duration of fixation on the events) exceeded the length of one whole familiarization trial for both agents (Experiment 1 and 2 physically intuitive/inefficient agent stimuli 22s, physically counterintuitive/efficient agent stimuli 22s; Experiment 3 & 4 physically intuitive/inefficient agent stimuli 20s, physically counterintuitive/efficient agent stimuli 15.5s). Secondly, we excluded the participants who did not watch the critical event in the test trials in which one agent took the reward. The agreement of the two coders was 99% of all trails (95/96). See *Participants* section for the number of excluded participants.

<u>Infants' visual exposure to the videos in the familiarization phase</u>

To (1) confirm that the infant attended to each video stimulus for both agents and (2) investigate whether the results of test phase was influenced by the length of visual exposure to the events in familiarization, we coded and analyzed the total duration of fixation towards each event in the familiarization phase. Duration of fixation at each trial during the familiarization phase was measured from the time point that a reward dropped into the ground to the point that the agent disappeared from the screen.

To achieve the first aim, we calculated the percentage of the total fixation of gaze duration for the first four trials. We plotted total duration of fixation for the final sample in Fig. S2. On average, infants looked at the familiarization stimuli >95% for first two trials of both events (presented to every participants). Thus, we consider that infants' exposure to the videos is sufficient to create a mental representation for the physically intuitive and physically counterintuitive events in familiarization.

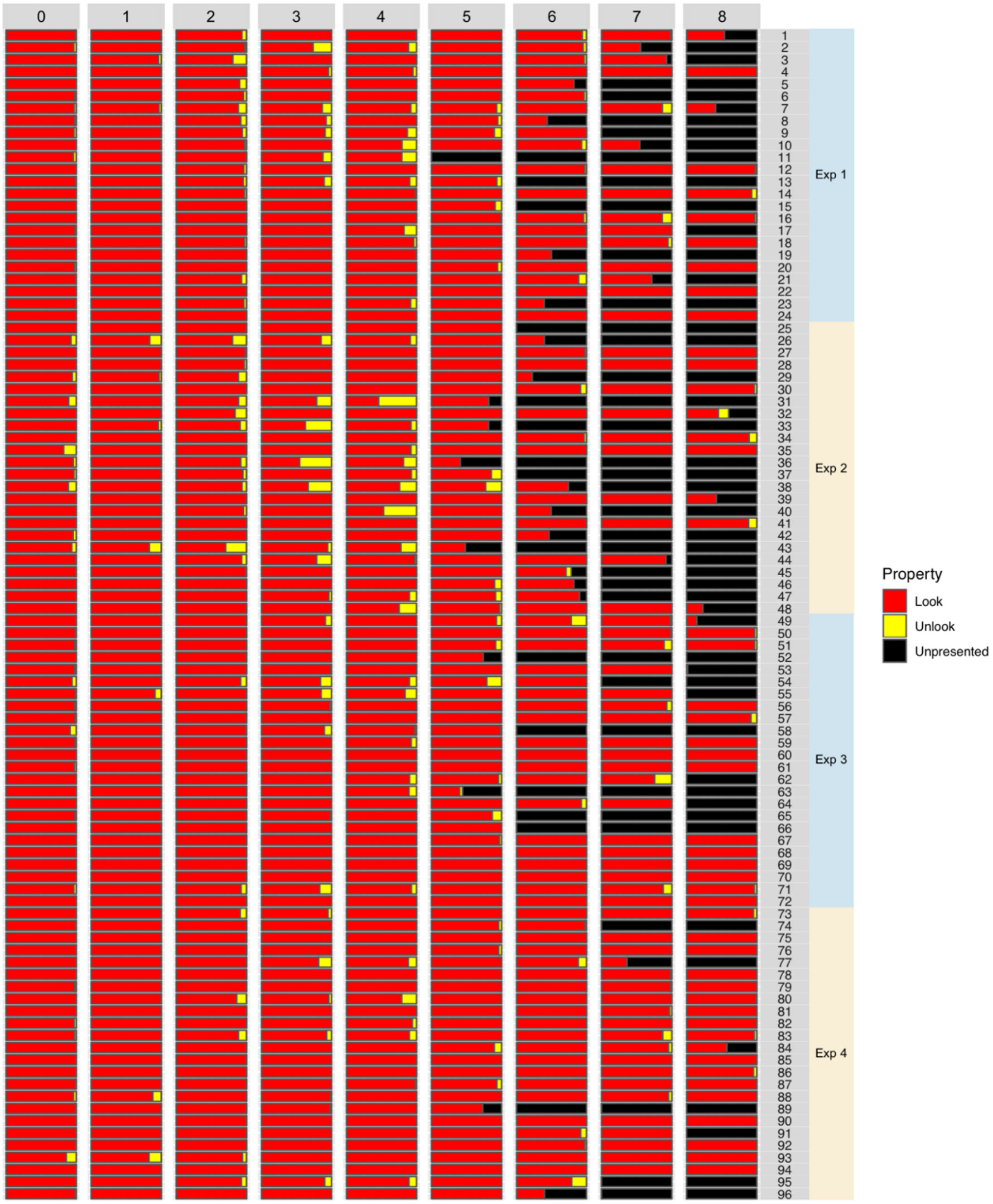

Fig. S2. Duration of fixation measurement for the familiarization. Each row represents participants, and each column represents the warm-up phase and a trial of the familiarization phase (i.e., “0”: the warm-up phase, “1-8”: trials of the familiarization). Each bar signifies 100 % of the whole trial (the transition between trials are not included). After the four trials (i.e., “1-4”), for participants looked

away 2 seconds or more, the familiarization phase was exterminated; the length exterminated was coloured black (include 2s for extermination criteria).

To achieve the second aim (testing whether the results of test phase was influenced by the length of visual exposure to the events in familiarization), we analyzed the total duration of gaze fixation during each event in the familiarization phase. Note that because the events were presented successively, and the presentation order was fixed, we predicted that infants would show longer total duration of fixation on the physically intuitive (or inefficient) events than the physically counterintuitive (or efficient) events. There were two reasons for this prediction. First, the current design was a departure from previous studies designed to measure differences in infants' looking time with respect to physically intuitive versus counterintuitive event [7–9]. In previous studies, events freeze and infants have enough time to watch the screen until they "lose interest" and look away. These studies find that it takes longer for infants to "lose interest" in the frozen counterintuitive events than the frozen intuitive events. In addition, infants generally continue to look at the frozen screen for > 20 seconds. In contrast, in the familiarization phase of the current study, events were presented one by one without the events freezing, making it possible to present a new event before infants "lost interest". Given this design, we did not predict that infants would look longer at the counterintuitive event. Second, the fixed presentation order may had led to a primacy effect, such that the intuitive (or inefficient) events always elicited longer total duration of fixation than the counterintuitive (or efficient) events, because the familiarization ended once infants had looked away from the screen for 2 seconds regardless of the properties of the events being presented.

The total duration of fixation was log-transformed for data analysis as a previous research has recommended [10]. Results showed that total duration of fixation in the familiarization phase with regard to both events consistently differed. As predicted, physically intuitive (or inefficient) events in the familiarization phase evinced longer total duration of fixation in all four experiments. A three-way ANOVA on total duration of fixation (*Table S1*) with event type (physically intuitive or inefficient/counterintuitive or

efficient event) as within-subject factor, and domain type (gravity/continuity) and experimental condition (manipulation/control) as between-subject factors revealed a main effect of event type [$F(1, 91) = 48.203$, $p < 0.001$, $\eta_p^2 = 0.346$], which was qualified by the interaction of the event type and domain [$F(1, 91) = 3.671$, $p = 0.009$, $\eta_p^2 = 0.072$]. All further post-hoc comparisons within domains showed that total duration of fixation was significantly longer toward physically intuitive (or inefficient) events than physically counterintuitive (or efficient) events (*ps* < 0.001).

Table S1. Descriptive statistics of the total duration of fixation in the familiarization

	physically intuitive (or inefficient) event				physically counterintuitive (or efficient) event			
	Exp 1	Exp 2	Exp 3	Exp 4	Exp 1	Exp 2	Exp 3	Exp 4
Mean	76.12 s	71.15 s	72.63 s	74.83 s	67.20 s	60.26 s	50.95 s	55.72 s
SD	12.79	15.03	10.73	8.49	16.35	19.08	12.83	9.55

Importantly, results did not show any significant effects related to experimental condition (the interaction of event type and experimental condition: [$F(1, 91) = 0.880$, $p = 0.351$, $\eta_p^2 = 0.010$], the interaction of event type, domain and experimental condition: [$F(1, 91) = 0.088$, $p = 0.768$, $\eta_p^2 = 0.001$]). Therefore, the effects of our finding that social dominance attribution only occurs in the experimental condition but not the control condition cannot be attributed to longer perceptual exposure to the physically intuitive (or inefficient) events in familiarization.

Overall analysis of all experiments (on looking time of the test phase)

We merged the data of all four experiments, and analyzed it to confirm that the effect appears only in experimental condition and that it is independent of domain. We used the log-transformed looking time for the statistically analyses [10]. Results of the ANOVA were shown in Table S2-4.

Table S2. Effects of within-subjects factor Test type on looking time in all experiments.

	Sum of Squares	df	Mean Square	F	p	η^2	η_p^2
Test type	1.108	1	1.108	3.486	0.065	0.012	0.037
Test type × Domain	0.035	1	0.035	0.110	0.741	0.000	0.001
Test type × Condition	2.984	1	2.984	9.391	0.003	0.032	0.093
Test type × Domain × Condition	0.100	1	0.100	0.313	0.577	0.001	0.003
Residual	29.236	92	0.318				

Note. Type III Sum of Squares

Table S3. Effects of between-subjects factor (Domain, Condition) on looking time in all experiments.

	Sum of Squares	df	Mean Square	F	p	η^2	η_p^2
Domain	0.081	1	0.081	0.129	0.720	0.001	0.001
Condition	0.001	1	0.001	0.001	0.988	0.000	0.000
Domain× Condition	1.868	1	1.868	2.986	0.087	0.031	0.031
Residual	57.563	92	0.626				

Note. Type III Sum of Squares

Table S4. Simple Main Effects of Test type by Condition on looking time in all experiments.

Condition	Sum of Squares	df	Mean Square	F	p
Control (Exp 2 & 4)	0.228	1	0.228	0.778	0.382
Experimental (Exp 1 & 3)	3.864	1	3.864	11.276	0.002

Note. Type III Sum of Squares

Figure 3 in the main article indicated that several data of looking time should be treated as outliers. We did not, prior to the experiment, plan to exclude outliers from the sample because, discarding data is considered to be detrimental to statistical efficiency [10]. We have applied logarithmic transformation to the data before statistical analysis to increase the validity of the analysis. However, to confirm that outliers did not influence

the findings, we further conducted an ANOVA on the data in which outliers were excluded (*Table S5-7*). The results indicated that whether or not the outliers were included as the dependent variables did not change the findings.

Table S5. Effects of within-subjects factor Test type on looking time (outliers excluded) in all experiments.

	Sum of Squares	df	Mean Square	F	p	η^2	η_p^2
Test type	0.859	1	0.859	2.898	0.092	0.010	0.032
Test type × Domain	0.030	1	0.030	0.101	0.751	0.000	0.001
Test type × Condition	2.564	1	2.564	8.644	0.004	0.031	0.090
Test type × Domain × Condition	0.100	1	0.100	0.313	0.577	0.001	0.003
Residual	25.806	87	0.297				

Note. Type III Sum of Squares

Table S6. Effects of between-subjects factor (Domain, Condition) on looking time (outliers excluded) in all experiments.

	Sum of Squares	df	Mean Square	F	p	η^2	η_p^2
Domain	0.004	1	0.004	0.007	0.934	0.000	0.000
Condition	0.016	1	0.016	0.027	0.869	0.000	0.000
Domain× Condition	2.195	1	2.195	3.648	0.059	0.040	0.040
Residual	52.348	87	0.602				

Note. Type III Sum of Squares

Table S7. Simple Main Effects of Test type by Condition on looking time (outliers excluded) in all experiments.

Condition	Sum of Squares	df	Mean Square	F	p
Control (Exp 2 & 4)	0.225	1	0.225	0.843	0.364
Experimental (Exp 1 & 3)	3.233	1	3.233	9.914	0.003

Note. Type III Sum of Squares

Exploratory analyses of the test phase

We used the log-transformed looking time for the statistically analyses [10]. For each experiment, we conducted exploratory analyses with ANOVA to rule out possible effects of the order of presentation of test trials (test order) and sex of participants (sex). Specifically, the looking time of the two test trials were compared with the property of the agent as the within-subject variables, and test order and sex as the between-subject variables (eta-squared η^2 and partial eta-squared η_p^2 were calculated for the effect size). Visual presentation of data were mainly conducted with R (https://www.r-project.org), and ANOVA was conducted with JASP (https://jasp-stats.org/) and jamovi (https://www.jamovi.org/). Below, we report the results of ANOVAs in all experiments.

Experiment 1 (domain: gravity, experimental condition)

A three-way ANOVA was performed on looking times with within-subject factor of test type (physically counterintuitive vs. intuitive agent dominant) and between-subject factors of test order (obtained the reward in the first vs. second test trial) and sex. A main effect of test outcome was found [$F(1, 20) = 5.36$, $p = 0.031$, $\eta_p^2 = 0.211$]: infants looked longer when the physically intuitive agent obtained the reward (*Table S8*). No other main effect or interaction was found [$ps > 0.14$, see *Table S8-9*]. Thus, neither the order of presentation of test trials nor sex of participants seemed to influence infants' evaluation of social dominance.

Table S8. Effects of within-subjects factor Test type (i.e., either the physically counterintuitive or physically intuitive agent obtained the reward in the test) on looking time in Experiment 1.

	Sum of Squares	df	Mean Square	F	p	η^2	η_p^2
Test type	1.413	1	1.413	5.357	0.031	0.090	0.211
Test type × Test order	0.023	1	0.023	0.086	0.773	0.001	0.004
Test type × Sex	0.109	1	0.109	0.415	0.527	0.007	0.020
Test type × Test order × Sex	0.043	1	0.043	0.163	0.690	0.003	0.008
Residual	5.277	20	0.264				

Table S8. Effects of within-subjects factor Test type (i.e., either the physically counterintuitive or physically intuitive agent obtained the reward in the test) on looking time in Experiment 1.

	Sum of Squares	df	Mean Square	F	p	η^2	η_p^2

Note. Type III Sum of Squares

Table S9. Effects of between-subjects factor (Test order, Sex) on looking time in Experiment 1.

	Sum of Squares	df	Mean Square	F	p	η^2	η_p^2
Test order	0.885	1	0.885	2.361	0.140	0.100	0.106
Sex	0.120	1	0.120	0.321	0.577	0.014	0.016
Test order × Sex	0.304	1	0.304	0.809	0.379	0.034	0.039
Residual	7.500	20	0.375				

Note. Type III Sum of Squares

Experiment 2 (domain: gravity, control condition)

As the previous experiment, the same three-way ANOVA was performed on looking times. No main effects and no interaction effects were found [*Ps* > 0.245, see *Table S10-11*], suggesting that neither the order of presentation of test trials nor sex of participants influenced infants' evaluation of social dominance.

Table S10. Effects of within-subjects factor Test type (i.e., either the efficient or inefficient agent obtained the reward in the test) on looking in Experiment 2.

	Sum of Squares	df	Mean Square	F	p	η^2	η_p^2
Test type	0.001	1	0.001	0.003	0.955	0.000	0.000
Test type × Test order	0.180	1	0.180	0.584	0.453	0.009	0.028
Test type × Sex	0.018	1	0.018	0.058	0.811	0.001	0.003
Test type × Test order × Sex	0.239	1	0.239	0.779	0.388	0.012	0.037
Residual	6.145	20	0.307				

Table S10. Effects of within-subjects factor Test type (i.e., either the efficient or inefficient agent obtained the reward in the test) on looking in Experiment 2.

	Sum of Squares	df	Mean Square	F	p	η^2	η_p^2

Note. Type III Sum of Squares

Table S11. Effects of between-subjects factor (Test order, Sex) on looking time in Experiment 2.

	Sum of Squares	df	Mean Square	F	p	η^2	η_p^2
Test order	0.099	1	0.099	0.152	0.701	0.007	0.008
Sex	0.001	1	0.001	0.001	0.984	0.000	0.000
Test order × Sex	0.937	1	0.937	1.435	0.245	0.066	0.067
Residual	13.058	20	0.653				

Note. Type III Sum of Squares

Experiment 3 (domain: continuity, experimental condition)

As the previous experiments, a three-way ANOVA was performed. A main effect of test outcome was found [$F(1, 20) = 5.536$, $p = 0.029$, $\eta_p^2 = 0.217$]: infants looked longer at test video when the physically intuitive agent obtained the reward. No other main effects or interaction were found [*p*s > 0.115, see *Table S12-13*]. Thus, neither the order of presentation of test trials nor sex of participants influenced infants' evaluation of social dominance.

Table S12. Effects of within-subjects factor Test type (i.e. either the physically counterintuitive or physically intuitive agent obtained the reward in the test) on looking time in Experiment 3.

	Sum of Squares	df	Mean Square	F	p	η^2	η_p^2
Test type	2.577	1	2.577	5.536	0.029	0.067	0.217
Test type × Test order	0.630	1	0.630	1.352	0.259	0.016	0.063
Test type × Sex	0.250	1	0.250	0.538	0.472	0.007	0.026

Table S12. Effects of within-subjects factor Test type (i.e. either the physically counterintuitive or physically intuitive agent obtained the reward in the test) on looking time in Experiment 3.

	Sum of Squares	df	Mean Square	F	p	η^2	η_p^2
Test type × Test order × Sex	0.043	1	0.043	0.092	0.765	0.001	0.005
Residual	9.312	20	0.466				

Note. Type III Sum of Squares

Table S13. Effects of between-subjects factor (Test order, Sex) on looking time in Experiment 3.

	Sum of Squares	df	Mean Square	F	p	η^2	η_p^2
Test order	0.001	1	0.001	0.001	0.973	0.000	0.000
Sex	2.921	1	2.921	2.711	0.115	0.115	0.119
Test order × Sex	0.935	1	0.935	0.868	0.363	0.037	0.042
Residual	21.556	20	1.078				

Note. Type III Sum of Squares

Experiment 4 (domain: continuity, control condition)

As the previous experiment, the same three-way ANOVA was performed on looking times. No main effects and no interaction effects were found (*ps* > 0.078, see *Table S14-15*), suggesting that neither the order of presentation of test trials nor sex of participants influenced infants' evaluation of social dominance.

Table S14. Effects of within-subjects factor Test type (i.e., either the efficient or inefficient agent obtained the reward in the test) on looking time in Experiment 4.

	Sum of Squares	df	Mean Square	F	p	η^2	η_p^2
Test type	0.248	1	0.248	0.903	0.353	0.014	0.043
Test type × Test order	0.852	1	0.852	3.101	0.094	0.049	0.134
Test type × Sex	0.947	1	0.947	3.448	0.078	0.055	0.147

Table S14. Effects of within-subjects factor Test type (i.e., either the efficient or inefficient agent obtained the reward in the test) on looking time in Experiment 4.

	Sum of Squares	df	Mean Square	F	p	η^2	η_p^2
Test type × Test order × Sex	0.013	1	0.013	0.047	0.831	0.001	0.002
Residual	5.492	20	0.275				

Note. Type III Sum of Squares

Table S15. Effects of between-subjects factor (Test order, Sex) on looking time in Experiment 4.

	Sum of Squares	df	Mean Square	F	p	η^2	η_p^2
Test order	0.008	1	0.008	0.016	0.900	0.001	0.001
Sex	0.386	1	0.386	0.828	0.374	0.040	0.040
Test order × Sex	0.013	1	0.013	0.029	0.867	0.001	0.001
Residual	9.333	20	0.467				

Note. Type III Sum of Squares

References:

1. Thomsen, L., Frankenhuis, W. E., Ingold-Smith, M. & Carey, S. Big and Mighty: Preverbal Infants Mentally Represent Social Dominance. *Science (80-.).* **331**, 477–480 (2011).
2. Mascaro, O. & Csibra, G. Representation of stable social dominance relations by human infants. *Proc. Natl. Acad. Sci.* **109**, 6862–6867 (2012).
3. Mascaro, O. & Csibra, G. Human Infants' Learning of Social Structures. *Psychol. Sci.* **25**, 250–255 (2014).
4. Pun, A., Birch, S. A. J. & Baron, A. S. Infants use relative numerical group size to infer social dominance. *Proc. Natl. Acad. Sci.* **113**, 2376–2381 (2016).
5. Cohen, J. A power primer. *Psychol. Bull.* **112**, 155 (1992).
6. Wiltermuth, S. S. & Heath, C. Synchrony and Cooperation. *Psychol. Sci.* **20**, 1–5 (2009).

7. Baillargeon, R. & Hanko-Summers, S. Is the top object adequately supported by the bottom object? young infants' understanding of support relations. *Cogn. Dev.* **5**, 29–53 (1990).
8. Baillargeon, R., Spelke, E. S. & Wasserman, S. Object permanence in five-month-old infants. *Cognition* **20**, 191–208 (1985).
9. Needham, A. & Baillargeon, R. Intuitions about support in 4.5-month-old infants. *Cognition* **47**, 121–148 (1993).
10. Csibra, G., Hernik, M., Mascaro, O., Tatone, D. & Lengyel, M. Statistical treatment of looking-time data. *Dev. Psychol.* **52**, 521–536 (2016).
